# Investigation into the sero-molecular prevalence of *Brucella melitensis* in small ruminants in districts Mohmand and Charsadda Khyber Pakhtunkhwa Pakistan

**DOI:** 10.1371/journal.pone.0315206

**Published:** 2025-02-07

**Authors:** Sohaib Ul Hassan, Farhan Anwar Khan, Muhammad Shuaib, Muhammad Shahid, Said Sajjad Ali Shah, Shahrood Ahmed Siddiqui, Anthony Pokoo-Aikins, Ayman A. Swelum

**Affiliations:** 1 College of Veterinary Sciences, Faculty of Animal Husbandry and Veterinary Sciences, The University of Agriculture, Peshawar, Khyber Pakhtunkhwa, Pakistan; 2 Key Laboratory of Animal Breeding, Reproduction and Molecular Design for Jiangsu Province, College of Animal Sciences and Technology, Yangzhou University, Yangzhou, China; 3 Directorate of Livestock and Dairy Development (Research Wing), Peshawar, Khyber Pakhtunkhwa, Pakistan; 4 Vaccine Production Unit, Livestock and Fisheries Department Government of Sindh, Sindh Tandojam, Pakistan; 5 US National Poultry Research Center, Toxicology and Mycotoxin Research Unit, USDA ARS, Athens, Georgia, United States of America; 6 Department of Animal Production, College of Food and Agriculture Sciences, King Saud University, Riyadh, Saudi Arabia; Beni Suef University Faculty of Veterinary Medicine, EGYPT

## Abstract

Globally, ruminants contribute largely to the livelihood and supply of quality food for human consumption. However, small ruminants face numerous problems, including infectious diseases, in lower- and middle-income countries (LMIC). Brucellosis is one of the important zoonotic diseases affecting the range of animals caused by Brucella species, including Brucella abortus and *Brucella mellitensis*. Although brucellosis caused by *B. mellitensis* in small ruminants has never been reported in the study areas, its zoonotic importance can never be underestimated. Therefore, this study was designed to investigate the sero-molecular prevalence of *B. mellitensis* in small ruminants in districts Mohmand and Charsadda of Khyber Pakhtunkhwa, Pakistan. A total of 400 blood samples were collected from sheep and goats (n = 200 from each species) and analyzed by Rose Bengal precipitation test (RBPT), the indirect enzyme-linked immunosorbent assay (*i*-ELISA) and polymerase chain reaction (PCR). The findings of the study indicated 13.5% and 7% of sheep while 12.5% and 12.5% of goat’s samples by RBPT and (i-ELISA) respectively. The species-specific PCR confirmed *B. abortus* in 70% of sheep samples and 37.5% of goat’s samples and *B. mellitensis* in 25% of sheep and 62.5% of goat’s samples by targeting IS711. The findings of the study concluded that *B. abortus* and *B. melitensis* were circulating in sheep and goats with a higher prevalence in the study areas. This study detected the presence of *B. mellitensis* for the first time in small ruminants in the study areas.

## Introduction

One of the important sectors of agriculture is livestock which is the pillar of the national economy of Pakistan. In 2019, 90.8 million cattle and buffaloes, 109.4 million small ruminants (sheep and goats), 1.1 million camels, and 6.1 million horses were estimated to comprise the nation’s livestock population. In Charsadda, there are 4.9 million sheep and 11.9 million goats, whereas in Mohmand, there are 4.1 million sheep and 6.8 million goats [1]. This livestock subsector contributed 11.7% of the country’s GDP and 60.6% of the value of the agriculture sector in 2019–2020 [2]. Pakistan is divided into seven administrative regions: Khyber Pakhtunkhwa (KPK), Balochistan, Sindh, Punjab, Gilgit-Baltistan, Azad Jammu and Kashmir (AJK), and the Islamabad Capital Territory (ICT). The country’s irrigated provinces of Sindh and Punjab are home to most cattle and buffaloes, whereas small ruminants are mainly found in the country’s dry regions. Brucellosis treatment and vaccination are preferably used for cattle rather than small ruminants [3]. Khyber Pakhtunkhwa is ranked third province in terms of both human and livestock populations [4].

Among many factors, production diseases the one which obstructs the economic return of small ruminants is brucellosis. Brucellosis is an infectious bacterial disease that is contagious and zoonotic of bacterial origin in cattle and small ruminants possessing a potential health alarming and economic importance worldwide. Brucellosis causes low reproductive efficiency characterized by repeated breeding [5]. The causative agents of brucellosis in small ruminants are *Brucella abortus*, *Brucella melitensis*, and *Brucella ovis* mainly characterized by abortion, stillbirth, placentitis, weak offspring with the development of yellowish, sticky layers on the placenta in females while in male orchitis, epididymitis, as well as inflammation of the joints and bursae, are affected [6,7]. In developing countries, it is still a major threat, serious systemic diseases caused by humans who are in contact with infected animals have seldom been explored and reported in human and domestic, wildlife, and aquatic animals [8]. In Pakistan, few studies have been conducted on brucellosis in small ruminants despite its zoonotic importance and heavy economic losses. Seroprevalence reported in different areas of Pakistan was reported as 4.4% [9], and 18.75% by [10] but to our knowledge, the data regarding small-ruminant brucellosis is scarce. It is reported that the prevalence of brucellosis is affected by many factors such as species, age, sex, diagnostic tool, environment, geological area, number of animals reared in the locality, and genetic makeup of the animal which act as tools for difference in results [11]. Many countries have adopted different control strategies based on the eradication of diseased animals detected by serological and diagnostic tests and other vaccine-based control methods [12]. Currently, three types of diagnostic tools are applied to identify *Brucella* species: conventional, serological, and molecular-based diagnostic procedures [13].

In most laboratories, blood and tissue cultures are considered the only choice for disease diagnosis but they are time-consuming and keep a huge risk of zoonosis for laboratory workers, especially [14,15]. Because *Brucella* is contagious and requires isolation and culture in biosafety level III or IV laboratories. The Rose Bengal precipitation test (RBPT), the Coombs test, the standard plate agglutination test (SPAT), the immunological capture test, and the enzyme-linked immunosorbent assay (ELISA) are common serological procedures for the diagnosis of brucellosis. Therefore, the present study is considered as the first step to achieve data regarding brucellosis in small ruminants. Thus, the objective of the study was to identify *Brucella melitensis* and *Brucella abortus* in small ruminants in districts Mohmand and Charsadda, Khyber Pakhtunkhwa using RBPT (Rose Bengal Plate test), *i-*ELISA (Indirect Enzyme Linked Immunosorbent Assay) & PCR (Polymerase Chine Reaction) techniques, and to study risk factors associated with brucellosis seropositivity.

## Materials and methods

### Study area for sample collection

Khyber Pakhtunkhwa (KPK) province is located to the northwest of Pakistan between 34°0’ N–71°19’. KPK bears a total livestock population of 21.62 million (15.14% of the total population of the country) [16]. The climate varies from very hot (38–43°C) in summer to moderately cold (2–4°C) in winter. Mohmand and Charsadda are in the west of the province.

### Sample size determination for this study

The number of samples for this study was determined according to the formula given by [17], which is based on the expected prevalence, desired absolute precision (5%), and confidence interval (95%). The following formula was used for the determination of the sample size.


$$nadj\;=\;\frac{N\times n}{N+n}$$

Where, N = total population

n =  sample size estimated through the following formula


$$n=\frac{Z^{2}\times Pexp\left(1-Pexp\right)}{d^{2}}$$

Where, Pexp =  prevalence expected

d = desired absolute precision.

Z = the value of z at a 95% confidence interval, which is 1.96.

By assuming the expected prevalence at 50% and desired absolute precision at 5%, the required sample size according to this formula would be.

n = 384.16∞384

### Sample strategies for study

The sample size was calculated assuming an expected prevalence of 50%, 95% confidence level, and 5% desired precision. This results in 400 samples, which are rounded up to 200 samples from each district. A total of 400 blood samples (200 each from sheep and goats) were collected randomly. Animals were restrained for blood collection without any anesthesia and/or analgesia. All animals were handled as per the guidelines of the Ethical Committee. A total of 3 mL to 5 mL of blood samples were collected aseptically from the jugular veins of sheep and goats. To maintain the integrity of red blood cells, disposable syringes were utilized to collect blood samples, which were then introduced into both Gel tubes and EDTA tubes without applying any pressure. Proper labeling was employed, and the blood samples were transported to the laboratory of the Centre for Microbiology and Bacteriology at the Veterinary Research Institute Peshawar for further processing. The blood samples were centrifuged, and serum was separated and stored at -20°C till tested. None of the animals had a history of vaccination against brucellosis. All farms visited produced milk and meat for domestic consumption and rarely for commercial purposes.

### Serological tests

#### Rose Bengal plate test (RBPT).

Serological pipettes, applicator, sticks, mechanical rotator, light, glass-slide plate, wax pencil, carbon paper, and magnifying glass are required for this procedure. Using the colored Rose Bengal Plate Test (RBPT) antigens in accordance with the 2014 OIE recommendation was the first step in the screening process. A total of 400 blood (200 sheep and 200 goats) were screened by RBPT according to the method described by [18] using the antigen, which was obtained from Veterinary Research Institute, Lahore, Pakistan. All the reagents were kept at room temperature for 30-50 minutes before starting the procedure. 30µl of each serum sample and Rose Bengal antigen was added and mixed gently on a clear glass-slide plate. The glass-slid plate was manually rotated in a clock and anticlockwise manner for four minutes and the reaction was observed after four minutes for the presence of agglutination [19]. Results were recorded as complete agglutination indicated positive, partial agglutination as doubtful, and lack of agglutination indicated a negative result.

#### Indirect Enzyme-linked immunosorbent assay (*i*-ELISA).

The sample sera were tested by ID Screen® (IDvet, Grabels, France) indirect ELISA for detection of brucellosis (*Brucella melitensis* and *Brucella abortus*) antibodies. The ELISA was performed, and the results were calculated as per the manufacturer’s recommendations. In short, all the reagent of the kit was brought to room temperature, and the wash solution (20%) was diluted to 1x, by adding 95ml of distilled water to 5ml of 20x solution for making of 100ml working solution. Four wells were assigned as controls, i.e., two wells for positive and two wells for negative samples. Firstly, to obtain a coated microplate and record the position of each sample. An equal volume of 190μl dilution buffer was added to all wells. After that 10μl of the negative control was added to the first well and 10μl of the positive control was added to the second well. Similarly, 10μl of the serum sample was added to the remaining well of the plate. The plate was incubated (Digital incubator, Korea) for 45 minutes at 21°C. The solutions were removed and each well three times with approximately 300μl of wash solution. After 100μl of the conjugate was added to each well, the plate was shaken for two minutes in the automatic shaker. The plate was covered with an aluminum sheet and incubation of the plate was at 21°C for 30 minutes. Again, the plate was washed with approximately 300μl of wash solution 3 times. An equal volume of 100μl of the substrate was added to each well. The plates will be incubated in the dark for 15 minutes. An equal volume of 100μl of the stop solution was added to all wells to stop the reaction. The OD value of the samples was measured through an ELISA reader (AMP Platos R II made by AMEDA Labordiagnostlk GmbH, Krenngasse Austria), recorded and controlled at 450nm. After measuring the OD value, the values were put in the formula and the results as according to the instructions of the kits.

#### Molecular identification.

All samples that tested positive on RBPT and *i*-ELISA were further confirmed by Polymerase Chain Reaction. For molecular diagnosis, *Brucella* DNA was amplified and detected by PCR using protocol [20]. Briefly, serum samples were used to obtain DNA by using the kit method (Nucleospin Blood, Germany) following manufacturer protocol. DNA concentration and quantification were obtained by using Nanodrop software on a Nanodrop attached to a computer screen (A260/280). After the confirmation of concentration and purification of DNA, a PCR assay was performed by using already reported primers, used by [21,22]. The PCR assay was performed to target the genus *Brucella* using the genus-specific primer (BCSP31 gene (BCSP31- PCR). The samples that were positive for genus-specific primers were subjected to species-specific primers (IS711B and IS711M) for PCR (Thermo Fisher Scientific). The primers used in the PCR reaction are given in Table 1. A total of 25µl reaction mixture was prepared by adding 4µl master mix (Tiangen Biotech, Beijing, China), with 0.5µl each forward and reverse primers (10pmol), 15µl nuclease-free water, and 5µl DNA template. The optimized conditions for amplification were initial denaturation at 95°C for 5 min., followed by 45 cycles of cyclic denaturation at 94°C for 1 min, 1 min. At 57°C for annealing and 1 min. At 72°C for cyclic extension, with a final extension step of 72°C for 7 min. After the thermocycler program, the amplified product was run on 1.5% agarose gel in gel electrophoresis for 40 minutes at 500ampher and 110 volts. Gel electrophoresis was used for documentation of PCR amplified fragments from extracted DNA of serum samples. 8µl PCR product with 2µl loading dye along with 100 bp ladder was visualized on 1.5% gel stained with 3µl ethidium bromide product and observed by Gel documentation system (Fast Gel, Germany) and record.

**Table 1:** PCR primers of Brucella genus (*B. abortus* and *B. melitensis*).

PCR	Primer	Sequence (5ʹ → 3ʹ)	Target	Reference
*Brucella* genus	Forward Reverse	TGGCTCGGTTGCCAATATCAA CGCGCTTGCCTTTCAGGTCTG	bcsp31	[21]
*B. melitensis*	Forward Reverse	AACAAGCGGCACCCCTAAAA CATGCGCTATGATCTGGTTACG	IS711	[22]
*B. abortus*	Forward Reverse	GCGGCTTTTCTATCACGGTATTC CATGCGCTATGATCTGGTTACG	IS711	

#### Statistical analysis.

The raw data collected from a pre-designed questionnaire for both sheep and goats were manually entered in a Microsoft Excel spreadsheet. The data were statistically analyzed using Statistical Package for Social Science(SPSS for Windows version 21, SPSS Inc., Chicago, IL, USA). A chi-square test was performed to check the statistical significance between categorical variables and the prevalence of brucellosis. The results were considered statistically significant if p ≤  0.05 at a 95% confidence interval. Receiver operating characteristics (ROC) curve analysis was performed to assess the diagnostic accuracy of different tests, i.e., RBPT and *i*-ELISA by considering PCR as the gold standard test, and the cross-tabulated 2 × 2 contingency table was used to analyze true positive (TP), true negative (TN), false positive (FP), and false negative (FN), and sensitivity and specificity were also determined. The area under the curve (AUC) values between 0.9–1 were considered as excellent, 0.8–0.9 good, 0.7–0.8 fair, 0.6–0.7 poor, and lower than 0.6 were interpreted as failed [23,24].

#### Ethics statement.

The study was approved by the ethical committee of the College of Veterinary Sciences (CVS), The University of Agriculture Peshawar, Khyber Pakhtunkhwa Pakistan (No.5011.CVS dated: 27/05/2021). In both districts (Mohmand and Charsadda), blood samples were collected from the animals with the help of local veterinary officials as per standard operation procedures of the Livestock and Dairy Development Department, Government of Khyber Pakhtunkhwa, Pakistan, and at the free will of the animal, owners to participate in the study. No discrimination was done against apparently healthy or affected animals. No animals were killed/harmed during the blood collection and restraining process. No follow-up was done on the animals after the study. Written consent of free will to participate in the study was not possible as most of the animal owners were illiterate.

## Results

**Fig 1:**
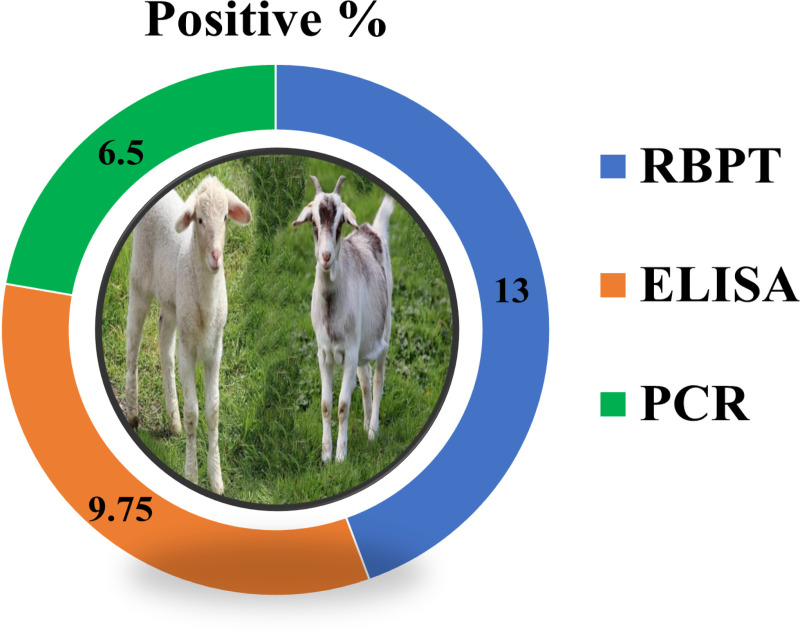
Prevalence of brucellosis in small ruminants in district Mohmand and Charsadda. Through RBPT, ELISA, and PCR.

The highest individual animal level seroprevalence (40%) was recorded in district Charsadda and (15.5%) in Mohmand. The overall seropositivity of brucellosis in sheep and goats was recorded at 13% by RBPT, 9.75% by *i*-ELISA, and 6.5% by PCR, respectively as shown in **“**Fig 1**”.**

**Fig 2:**
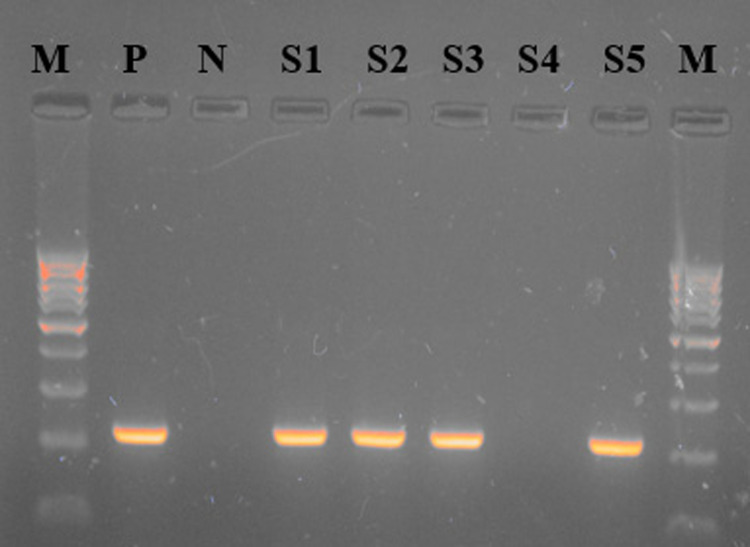
Gel picture showing 100 bp DNA ladder (M) and Brucella genus positive samples having amplicon size of 223 bp (P = Positive control, **N** =  Negative control and positive samples (S1, S2, S3, S5 while negative sample is S4).

**Fig 3:**
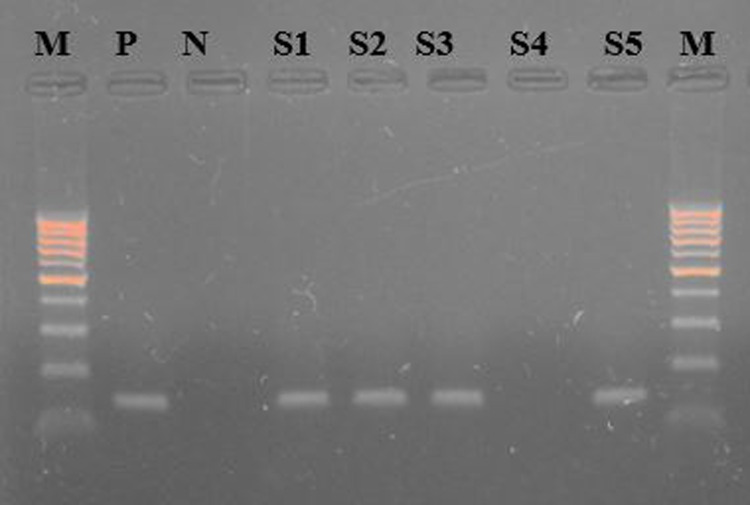
Gel picture showing 100 bp DNA ladder (M) and Brucella abortus positive samples having amplicon size of 133 bp (P = Positive control, **N** =  Negative control and positive samples (S1, S2, S3, S5 while negative sample is S4).

**Fig 4:**
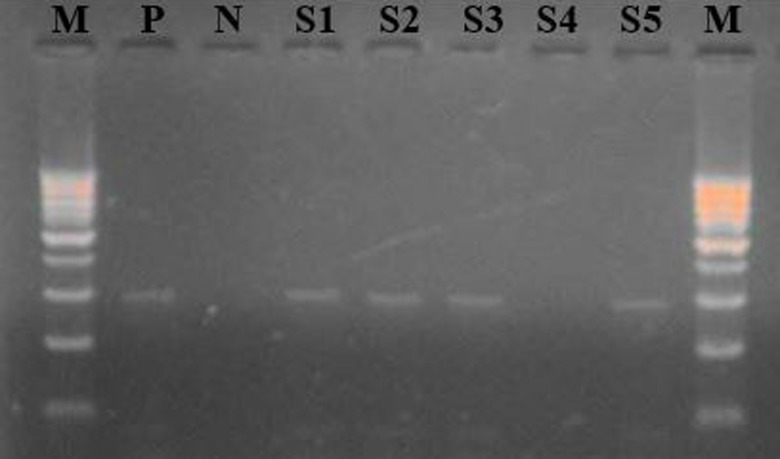
Gel picture showing 100 bp DNA ladder (M) and Brucella melitensis positive samples having amplicon size of 279 bp (P = Positive control, **N** =  Negative control and positive samples (S1, S2, S3, S5 while negative sample is S4).

The data presented in Table 2 depicts the seroprevalence of brucellosis in both districts Muhammad and Charsadda using RBPT, *i*-ELISA, and PCR. The RBPT results showed a higher prevalence of brucellosis in district Charsadda at 19% followed by district Mohmand with a prevalence rate of 4%. The *i-*ELISA results showed the prevalence of brucellosis was 12.5% and 7% while PCR results showed 8.5% and 4.5% in both districts Charsadda and Mohmand, respectively. The results of RBPT were significant (p ≤  0.05) statistically while the results of *i*-ELISA and PCR were non-significant in both districts. The seroprevalence among species in both districts through RBPT, *i*-ELISA, and PCR were 12%, 7.25%, and 7.2% respectively. The results of RBPT and PCR were statistically non-significant while the results of *i*-ELISA were significant (P ≤  0.05). The results in **“**Table 2**”** also show population-wise seroprevalence of brucellosis in small ruminants. The male population shows the prevalence among the flock having more than and less than 10 in number on RBPT was 5.4 and 13.8%, *i*-ELISA 9.09 and 10% and PCR was 6.4 and 6.5%, respectively. The results of the male population were only significant (P ≤  0.05) on RBPT. The female population shows a prevalence rate among the flock having more than and less than 80 in number on RBPT was 6.1 and 15.9%, *i*-ELISA 7.2 and 11.8% and PCR was 5.5 and 7.3%, respectively. The results of the female population were only significant (P ≤  0.05) on RBPT. Herd size was recorded significantly (P ≤ 0.05) through RBPT 6.1 and 15.9%, while non-significant on *i-*ELISA 7.18 and 11.87% and PCR 5.5 and 7.3%, respectively. The seroprevalence of season in summer and winter on RBPT was 8.1 and 38%, *i*-ELISA 24.98 and 14%, and PCR 16.9 and 9.1%, respectively. The results were statistically significant on RBPT, *i*-ELISA, and PCR. The seroprevalence of brucellosis among sex-wise was analyzed by males and females. The results of males and females on RBPT were 11.5 and 14.9%, i-ELISA was 17.02 and 8.78, and PCR was 12.7 and 5.6%, respectively. The results for RBPT, *i*-ELISA, and PCR were non-significant. The seroprevalence of age-wise among young and adults on RBPT 9.8 and 15.4%, *i*-ELISA 5.8 and 16.4%, and PCR 3.9 and 10.3%, respectively. The results were statistically significant for *i*-ELISA and PCR whereas for RBPT was non-significance. The seroprevalence of parity among less than and more than 5 for brucellosis on RBPT 11.8 and 10.1%, *i*-ELISA 8.2 and 9.4%, and PCR 4.1 and 7.6%, respectively. The results were statistically non-significance for RBPT, *i*-ELISA, and PCR. The seroprevalence of brucellosis in small ruminants among aborted and non-aborted on RBPT 33.3 and 4.4%, *i*-ELISA 29.6 and 2.55%, and PCR 22.2 and 0.7%. The results were statistically significant (<0.05) on RBPT, *i*-ELISA and PCR. Examining the occurrence of brucellosis in small ruminants, all collected samples were analyzed through RBPT and indirect ELISA for initial screening followed by PCR for confirmatory diagnosis of the disease. Samples positive on PCR give amplicon sizes of 223 bp for the *Brucella* genus while *B. abortus and B. melitensis* give amplicon sizes of 133 bp and 279 bp as shown in “Fig 2, 3 and 4**, respectively”** (S2, S3 and S4 Fig) respectively. The diagnostic techniques-wise prevalence of brucellosis in small ruminants in district Mohmand through RBPT, *i*-ELISA, and PCR were shown in **“**Table 3**”**. The results of RBPT shows in sheep were 20% (20/100) while in goats were 16% (16/100). The seroprevalence of sheep and goats on *i*-ELISA was 10% (10/100) and 15%% (15/100), respectively. The results of PCR for sheep and goats were 30% (9/30) and 25% (8/31), respectively. The results on PCR for *B. abortus* for sheep and goats were 60% (6/9) and 25% (2/8) while the results on PCR for *B. melitensis* for sheep and goats were 30% (3/9) and 75% (6/8). The seroprevalence of brucellosis in small ruminants in district Charsadda through RBPT, *i*-ELISA, and PCR were shown in **“**Table 4**”**. The results of RBPT show that sheep were 7% (7/100) while goats were 9% (9/100). The seroprevalence of sheep and goats on *i*-ELISA was 4% (4/100) and 10%% (10/100), respectively. The PCR results for sheep and goats were 45% (5/11) and 21.1% (4/19), respectively. The results on PCR for *B. abortus* for sheep and goats were 80% (4/5) and 50% (2/4) while the results on PCR for *B. melitensis* for sheep and goats were 20% (1/5) and 50% (2/4).

**Table 2:** Detection of brucellosis in small ruminants through RBPT, *i*-ELISA, PCR and its association with districts, species, male population, female population, herd size, season, sex, age, parity, and abortion.

Variable	Category	RBPT		*i-*ELISA		PCR	
		Prevalence (%)	P-Value	Prevalence (%)	P-Value	Prevalence (%)	P-Value
District	Mohmand (n = 200)	4	0.00 *	7	0.64	4.5	0.1
	Charsadda (n = 200)	19		12.5		8.5	
Species	Sheep (200)	12	0.80	7.25	0.04 *	7.2	0.5
	Goat (200)	11		12.07		5.7	
Male Population	<10	13.8	0.02 *	10	0.784	6.5	0.94
	>10	5.4		9.09		6.4	
Female Population	<80	15.9	0.02 *	11.8	0.123	7.3	0.48
	>80	6.1		7.2		5.5	
Herd Size	<80	15.9	0.02 *	11.87	0.116	7.3	0.47
	>80	6.1		7.18		5.5	
Season	Summer	8.1	0.00 *	14	0.00 *	9.1	0.01 *
	Winter	38		24.98		16.9	
Sex	Male	11.5	0.438	17.02	0.07	12.7	0.06
	Female	14.9		8.78		5.6	
Age	Young	9.8	0.1	5.8	0.00 *	3.9	0.02 *
	Adult	15.4		16.4		10.3	
Parity	<5	11.8	0.8	8.2	0.8	4.1	0.35
	>5	10.1		9.4		7.6	
Abortion	Yes	33.3	0.00 *	29.6	0.00 *	22.2	0.01 *
	No	4.4		2.5		0.7	

*Indicates significance at P-value < 0.05.

*Male goats and sheep aged less than or equal to one year and female animals that had not yet given birth were included in the younger age group.

**Table 3:** Diagnostic techniques wise prevalence of brucellosis in district Mohmand.

Sheep				Goats		
**Diagnostic Technique**	**Total samples**	**positive samples**	**Positive percentage**	**Total samples**	**positive samples**	**Positive percentage**
RBPT	100	20	20%	100	16	16%
I-ELISA	100	10	10%	100	15	15%
PCR	30	9	30%	31	8	25%
PCR *(B. abortus*)	9	6	60%	8	2	25%
PCR *(B. melitensis*)	9	3	30%	8	6	75%

**Table 4:** Diagnostic techniques wise prevalence of brucellosis in district Charsadda.

Sheep				Goats		
**Diagnostic Technique**	**Total samples**	**positive samples**	**Positive percentage**	**Total samples**	**positive samples**	**Positive percentage**
RBPT	100	7	7%	100	9	9%
I-ELISA	100	4	4%	100	10	10%
PCR	11	5	45%	19	4	21.1%
PCR (*B. abortus*)	5	4	80%	4	2	50%
PCR (*B. melitensis*)	5	1	20%	4	2	50%

### The performance characteristics of RBPT and I-ELISA using PCR as a gold standard

**Fig 5:**
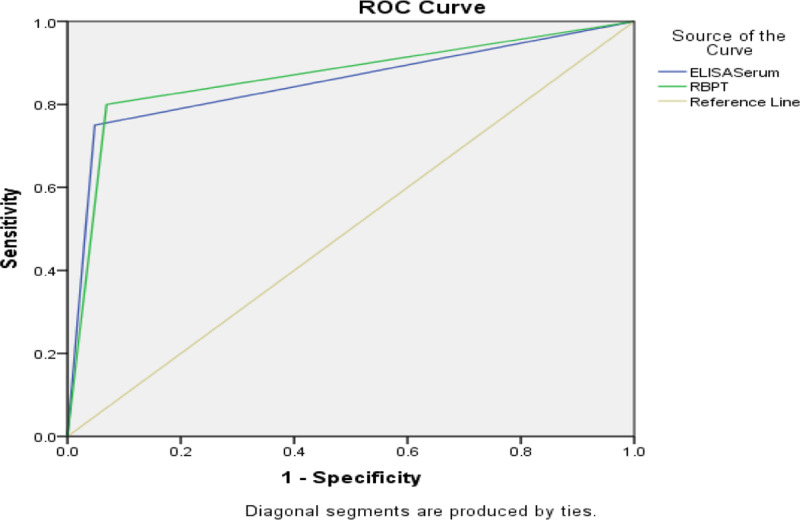
ROC curve for RBPT, SPAT, and ELISA while considering PCR as a reference test.


The accuracy of different diagnostic tests, i.e., RBPT and *i*-ELISA was determined by considering PCR as a gold standard test. The sensitivity for RBPT and *i*-ELISA was 80 and 75% while the specificity was 93.1 and 95% for RBPT and *i*-ELISA according to the formula, respectively. The positive predictive value (PPV) of RBPT and *i*-ELISA was 41.03 and 48.39%, whereas the negative predictive value (NPV) was 98.73 and 98.45% for RBPT and *i*-ELISA, respectively. The area under the ROC curve (AUC) for RBPT and *i*-ELISA was 0.865 and 0. 851, which were interpreted as moderated for RBPT and *i*-ELISA as shown in **“**Table 5**”** and **“**Fig 5**”.** $Sensitivity=\frac{True\;positive\;\;\left(a\right)}{True\;positive\;\;\left(a\right)+False\;positive\;\;\left(c\right)}\times 100$

**Table 5:** The Performance Characteristics of RBPT and ELISA using PCR as a Gold Standard.

Test	Sensitivity (%)	Specificity (%)	PPV (%)	NPV (%)	AUC
RBPT	80	93.1	41.03	98.73	0.865
I-ELISA	75	95	48.39	98.45	0.851

Note. RBPT Rose Bengal Plate Test, SPAT: Serum Plate Agglutination Test, PPV: Positive Predictive Value, NPV: Negative Predictive Value, AUC: Area Under Curve.

$$Specificity=\frac{True\;\;negative\;\;\left(d\right)}{True\;\;negative\;\;\left(d\right)+False\;positive\;\;\left(b\right)}\times 100$$

## Discussion

Brucellosis is an important disease that causes abortion in naturally infected small ruminants and is of great public health concern in many countries, including Pakistan [25]. In the study area, improper abortion management, common grazing lands, sharing watering points, keeping of animals together, and sharing of breeding ram/buck are the common practices observed. The situation is further provoked by poor farmers’ perception and awareness of disease transmission and abortion management. This agrees with the findings of [26] in Egypt and [27] in the Somali region, Ethiopia. Particularly in underdeveloped countries, community knowledge is essential for maintaining hygienic practices in rearing, raising, and breeding small ruminants to prevent diseased animals from being transferred to flocks free of disease [28]. The study highlights the importance of using appropriate serological and molecular tests for detecting and controlling brucellosis in small ruminants, thereby facilitating effective eradication and monitoring programs. Isolation and identification of organisms are the diagnosis gold standard [29], but it is cumbersome, takes several days to weeks, and poses a higher risk to laboratory personnel. Hence, the diagnosis of brucellosis largely depends on the use of two or more tests to confirm infection [30]. RBPT are current screening test, and *i*-ELISA is used as a confirmatory test for *B. abortus* and *B. melitensis* infection in small ruminants [31,32]

The overall occurrence of brucellosis in small ruminants in the present study was 13% by RBPT, 9.75% by *i*-ELISA, and 6.5% by PCR, respectively. These results were almost similar to the findings of [33,34]. While comparing different diagnostic tests, a higher number of positive cases were detected through RBPT as compared to *i*-ELISA, and possibly this might be because RBPT detects both IgM and IgG whereas *i*-ELISA detects IgM only [30]. The overall prevalence of brucellosis through *i*-ELISA in small ruminants was in line with [35]. Several studies reported that *i*-ELISA is more sensitive than conventional tests [36,37]. The higher sensitivity of *i*-ELISA due to its recognition of cytosolic antigen S-LPS fragments may decrease cross-reaction with other Gram-negative bacteria [38–40]. Other bacteria share similar epitopes with *Brucella* [41]. The prevalence of *Brucella* through PCR was higher in sheep as compared to goats, respectively. PCR detects DNA, which may be present in small amounts in serum samples. Alternatively, the titer in serum may be undetectable, but the amount of DNA present in the sample may be sufficient for PCR detection. As little as 5fg of DNA can be detected using PCR [42]. PCR is more dependable and sensitive due to its ability for antigenic detection than antibody detection [43]. In the present study, the PCR has shown more sensitivity to detect antibodies against *B. abortus* and *B. melitensis* as compared to other serological tests. This finding is by the findings of [18,44,45] who also reported PCR as more sensitive than other conventional assays.

The statistical analysis with risk factors showed that seropositivity to *Brucella* infection was significantly higher in females compared to males and this statement is supported by the findings of various researchers across the world [46]. [47] has stated that males are less susceptible to *Brucella* infection as compared to female animals, because of the absence of erythritol. [46] stated that erythritol is not present in the reproductive organs of males, less prone to infection. The most likely causes for the high occurrence in males in the present study were that most brucellosis-infected animals have reached sexual maturity, and the farmer uses infected male animals for breeding purposes due to which the disease spread in the flock. The incidence of brucellosis in males in the present study was in agreement with [25] that the owner of livestock does not cull or emplaced brucellosis-infected animals, instead utilizing them for breeding purposes or selling them to other ranchers, which is the most likely. Young animals are less susceptible to disease due to the growth and multiplication of *Brucella*, stimulated by erythritol and sex hormones, which increase with sexual maturation and age [48,49]. Additionally, this higher level of seroprevalence of brucellosis among older animals may be attributed to the sexual development of animals with the propelling age[48,50]. The higher level of seroprevalence in adult stock has been endorsed in other studies as well [1]. The observed differences in seropositivity between species may be due to goats’ susceptibility to *Brucella melitensis* due to their longer excretion period, reducing the spread of the disease among sheep flocks [6]. This is consistent with the finding of [51] who reported 11.76 times more risk in goats than sheep. The study found that larger flock of sheep and goats are more susceptible to disease, possibly due to poor local herd management, and no vaccination, and suggests that larger flock sizes are more susceptible to disease spread. This is comparable to the findings of [52], and [53] who reported higher seroprevalence in larger flocks than the smaller ones. Larger herds with more animal movements and intensive management practices increase the risk of disease spread due to higher exposure, contact during watering and feeding, and closer contact between animals and their environment [52,54]. The study revealed that animals with and without abortion history were significantly at risk for small-ruminant seropositivity, as per the statistical analysis. Reproductive loss due to abortion, birth of weak offspring, and infertility are recorded as the common clinical signs of brucellosis in natural hosts [55]. About 55% of the respondents determined that abortion in their flocks is one of the production problems listed. In the present study herds with a history of abortion were more likely to be positive than flocks with no previous abortion history. [56] from Uganda have also reported that herds with a history of abortion were 3.5 times more likely to have positive reactors. Similarly, [57] more positive reactors in animals with a history of abortion have also been reported in Ethiopia. Abortion in the third trimester of pregnancy is the most common symptom of brucellosis in breeding animals [58]. While comparing the prevalence of brucellosis in districts Mohmand and Charsadda, brucellosis was higher in district Charsadda. The high prevalence of brucellosis in sheep and goats in the Charsadda district may be due to differences in animal management and production systems between rural and farm areas, as well as the influx of nomads and the difficulty of finding positively tested animals among poor livestock farmers, potentially spreading the disease.

By comparing the results of serological tests used in this study with that of PCR as a gold standard test to find the sensitivity, specificity, Positive Predictive Value (PPV), Negative Predictive Value (NPV), and Area Under Curve (AUC). RBPT was found to be more sensitive than *i*-ELISA because the RBPT detects both IgG and IgM antibodies to *Brucella* infection in small ruminants [30]. IgM antibodies are present in the early stages of infection, making RBPT more sensitive for detecting recent or acute infections [59]. RBPT is a simple, rapid, and inexpensive test that can be performed in a short time. This simplicity reduces the chances of technical errors, which can increase its sensitivity [60]. RBPT uses a standardized antigen preparation, which ensures consistent results and increases sensitivity. RBPT is less prone to interference from other antibodies or serum components, which can reduce false negatives and increase sensitivity [61]. The specificity of *i*-ELISA was higher than RBPT. Firstly, *i*-ELISA detects IgG antibodies, which are more specific to *Brucella* infection than the IgM antibodies detected by RBPT. IgG antibodies take longer to develop, indicating a more established infection [30]. Secondly, *i*-ELISA uses a highly specific antigen, often a purified protein or lipopolysaccharide, which reduces cross-reactivity with other bacteria or antibodies [62]. Thirdly, *i*-ELISA reagents are highly standardized, minimizing variations in test performance and reducing false positives. *i*-ELISA provides quantitative results, allowing for a more accurate assessment of antibody levels and reducing the risk of false positives [63]. Fourthly, *i*-ELISA is less susceptible to interference from other serum components or antibodies, reducing false positives. *i*-ELISA has been shown to have higher diagnostic accuracy than RBPT, especially in areas with low brucellosis prevalence. *i*-ELISA can detect chronic infections [62], while RBPT may be missed due to its focus on IgM antibodies. *i*-ELISA is less dependent on operator expertise, reducing the risk of human error and increasing specificity [64] while RBPT is a useful screening test, *i*-ELISA is often preferred for confirmatory testing due to its higher specificity [65]. However, both tests have their place in brucellosis diagnosis, and the choice of test depends on the specific context and diagnostic needs.

## Supporting information

S1 Fig Un cropped gel picture. 1:Un cropped gel picture showing 100 bp DNA ladder (M) and Brucella genus positive samples having amplicon size of 223 bp (P = Positive control, N =  Negative control and positive samples (S1, S2, S3, S5 while negative sample is S4). 2: Un cropped gel picture showing 100 bp DNA ladder (M) and *Brucella abortus* positive samples having amplicon size of 133 bp (P = Positive control, N =  Negative control and positive samples (S1, S2, S3, S5 while negative sample is S4). 3: Un cropped gel picture showing 100 bp DNA ladder (M) and *Brucella melitensis* positive samples having amplicon size of 279 bp (P = Positive control, N =  Negative control and positive samples (S1, S2, S3, S5 while negative sample is S4).
(PDF)
