# Supplementary material for: Investigation into the sero-molecular prevalence of Brucella melitensis in small ruminants in districts Mohmand and Charsadda Khyber Pakhtunkhwa Pakistan
Source: PLoS One. 2025 Feb 7;20(2):e0315206. doi: 10.1371/journal.pone.0315206 (PMC11805427; doi:10.1371/journal.pone.0315206)
Supplement: S1 Fig — 1:Un cropped gel picture showing 100 bp DNA ladder (M) and Brucella genus positive samples having amplicon size of 223 bp (P = Positive control, N = Negative control and positive samples (S1, S2, S3, S5 while negative sample is S4). 2: Un cropped gel picture showing 100 bp DNA ladder (M) and Brucella abortus positive samples having amplicon size of 133 bp (P = Positive control, N = Negative control and positive samples (S1, S2, S3, S5 while negative sample is S4). 3: Un cropped gel picture showing 100 bp DNA ladder (M) and Brucella melitensis positive samples having amplicon size of 279 bp (P = Positive control, N = Negative control and positive samples (S1, S2, S3, S5 while negative sample is S4). (PDF) [file pone.0315206.s001.pdf]

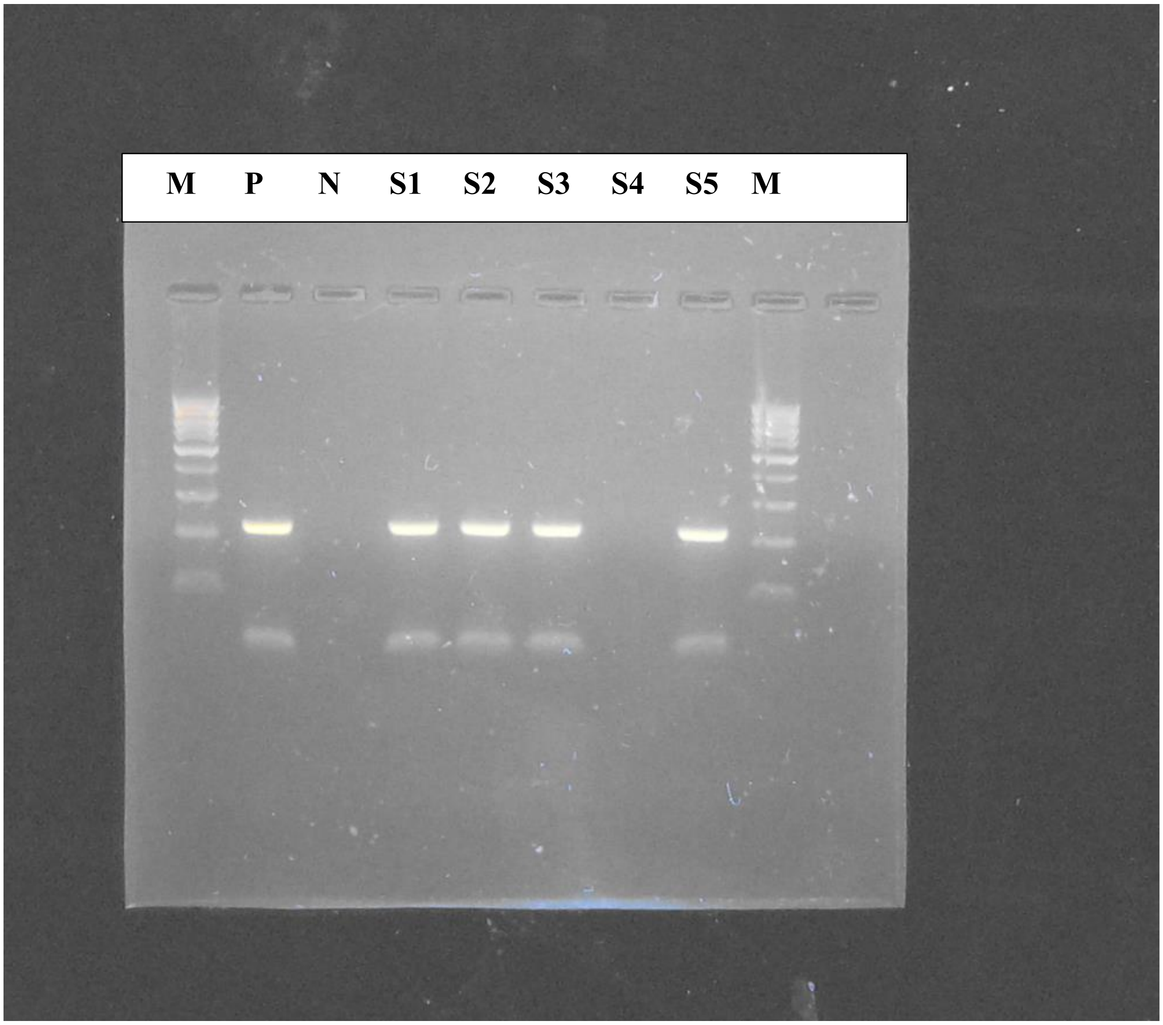


**Figure 2:** Gel picture showing 100bp DNA ladder (M) and *Brucella* genus positive samples having amplicon size of 223bp (P= Positive control, N = Negative control and positive samples (S1, S2, S3, S5 while negative sample is S4).

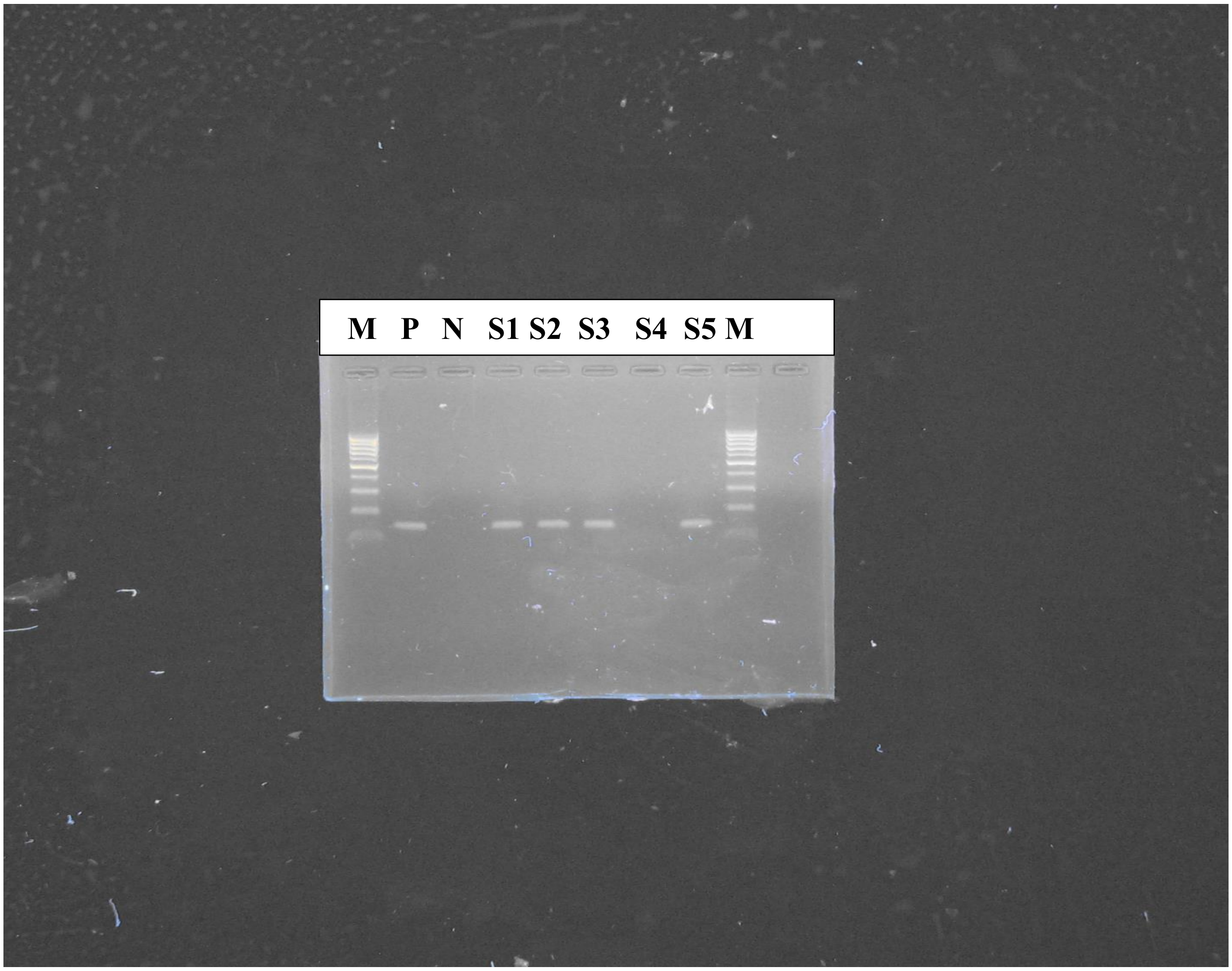


**Figure 3:** Gel picture showing 100bp DNA ladder (M) and *Brucella abortus* positive samples having amplicon size of 133bp (P= Positive control, N = Negative control and positive samples (S1, S2, S3, S5 while negative sample is S4).

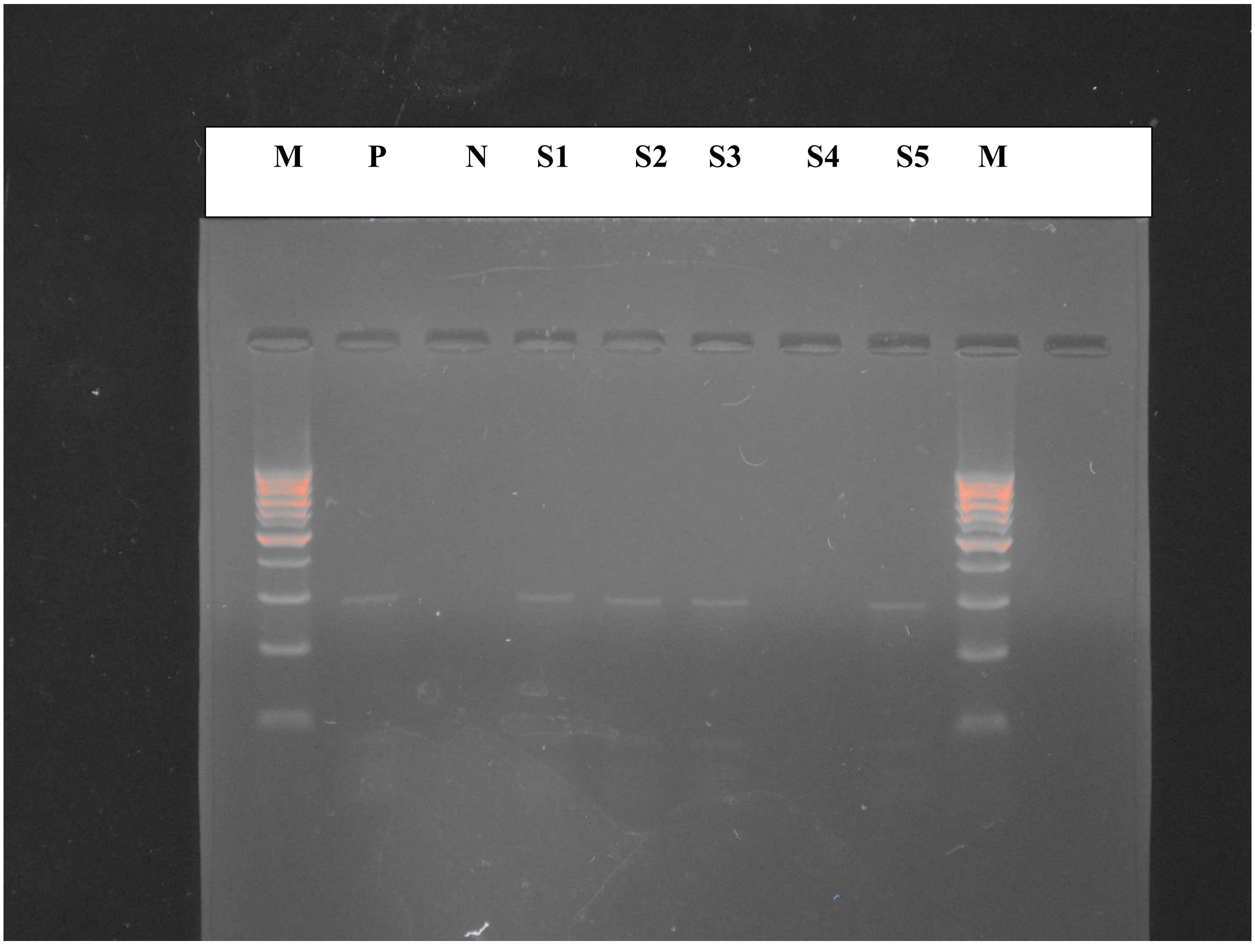


**Figure 4:** Gel picture showing 100bp DNA ladder (M) and *Brucella melitensis* positive samples having amplicon size of 279bp (P= Positive control, N = Negative control and positive samples (S1, S2, S3, S5 while negative sample is S4).
